# The Trajectory of Motor Deterioration to Death in Parkinson's Disease

**DOI:** 10.3389/fneur.2021.670567

**Published:** 2021-08-18

**Authors:** Sabrina Poonja, Janis Miyasaki, Xilai Fu, Richard Camicioli, Tina Sang, Yan Yuan, Fang Ba

**Affiliations:** ^1^Division of Neurology, Department of Medicine, University of Alberta, Edmonton, AB, Canada; ^2^School of Public Health, University of Alberta, Edmonton, AB, Canada; ^3^Department of Science, University of Alberta, Edmonton, AB, Canada

**Keywords:** Parkinson's disease, trajectory, UPDRS, terminal decline, palliative care

## Abstract

**Background:** Motor progression varies even among those with a single diagnosis such as Parkinson's disease (PD) and little is known about the trajectory of motor signs prior to death. Understanding deterioration patterns may help clinicians counsel patients and proactively plan interdisciplinary care, including palliative care. The objective of this study was to examine and describe Unified Parkinson's Disease Rating Scale motor score (UPDRS-III) trajectories at the end of life in PD.

**Methods:** A retrospective chart review was performed for deceased PD patients who attended the Parkinson and Movement Disorders Program at the University of Alberta for at least 5 years between 1999 and 2018. UPDRS-III scores were recorded for all visits. Trajectory patterns were visualized with Loess curves stratified by sex and age at diagnosis. Piecewise linear models were used to individually model the UPDRS-III scores, and the trajectories obtained were clustered based on their features.

**Results:** Among the 202 charts reviewed, 84 meeting inclusion criteria were analyzed. The UPDRS-III increased over time regardless of sex and age. Distinct trajectory variations present in PD (e.g., Consistent Deterioration, Stability-Deterioration, Improvement-Deterioration, Deterioration-Improvement-Deterioration) were identified. Twenty-five percent of the patients were classified as Undetermined/Irregular trajectories. In addition, regardless of trajectory type, many patients experienced a steep increase in UPDRS-III approaching death. Those with disease diagnosis after age 65 years had a shorter survival time, compared to PD patients with a younger age of onset.

**Conclusion:** Our study identified dominant types of motor trajectory in PD that can help clinicians understand their patients' course of illness. This information can help counsel patients regarding the variability in motor deterioration and should alert physicians to recognize a terminal decline. Age of disease onset was correlated with survival time.

## Introduction

Multiple factors influence the progression and trajectory of Parkinson's disease (PD). Studies on the natural history of PD revealed that despite all advances in the symptomatic management with new pharmacologic agents and technologies for PD, the progression of motor disability is inexorable, adding to patients and caregivers' burden, especially at the end of life (1–3). This encompasses disability caused by increasing severity of motor signs over time, development of motor complications, and poorly levodopa responsive axial motor signs, including dysarthria, dysphagia, postural instability, and freezing of gait (4, 5). In addition, non-motor symptoms increase in number and severity throughout the course of disease and in particular, neuropsychiatric complications of PD can be burdensome (6, 7).

PD is the most common parkinsonian condition. The prevalence is about 1% in people over the age of 60 years (8), and the reported incidence ranges from 8 to 18 per 100,000 person-years (9). The neuropathological hallmarks of PD are neuronal loss in the substantia nigra, which leads to striatal dopamine deficiency, and intracellular inclusions containing aggregates of α-synuclein (10). Levodopa and other dopamine enhancing agents increase the synaptic dopamine concentration and/or postsynaptic receptor binding, and therefore improve motor symptoms, especially early in the disease course. PD usually carries a better prognosis than the other atypical parkinsonian syndromes (8). Illnesses such as progressive supranuclear palsy, multiple system atrophy, and corticobasal syndrome have less response to treatment and usually progress more rapidly (11, 12). However, although considered a slowly progressive disease, there is marked heterogeneity in PD disease progression. PD motor phenotype is indicative of prognosis. The postural instability/gait difficulty (PIGD) phenotype usually has a poorer response to dopaminergic treatment and a worse prognosis than the tremor dominant phenotype (13).

Since PD is a progressive neurodegenerative condition, and the disease trajectory can vary, it is important to have a better understanding of the patterns of disease course and deterioration in later stages to help clinicians counsel patients and plan interdisciplinary care, including palliative care referrals, accordingly. The objective of this study was to examine and describe UPDRS-III trajectories at the end of life in PD. These data can help patients, families and clinicians understand potential progression trajectories in advanced illness. Since most research has focused on early and mid-stage PD, the end of life has been largely neglected. Following PD patients until their death is unusual in many neurology practices. This data can also help patients, families and clinicians identify terminal motor decline as a trigger for palliative care involvement. More importantly, this study can provide clinicians, patients and families with realistic expectations when making important goals of care decisions.

## Materials and Methods

### Study Subjects

**Figure 1:**
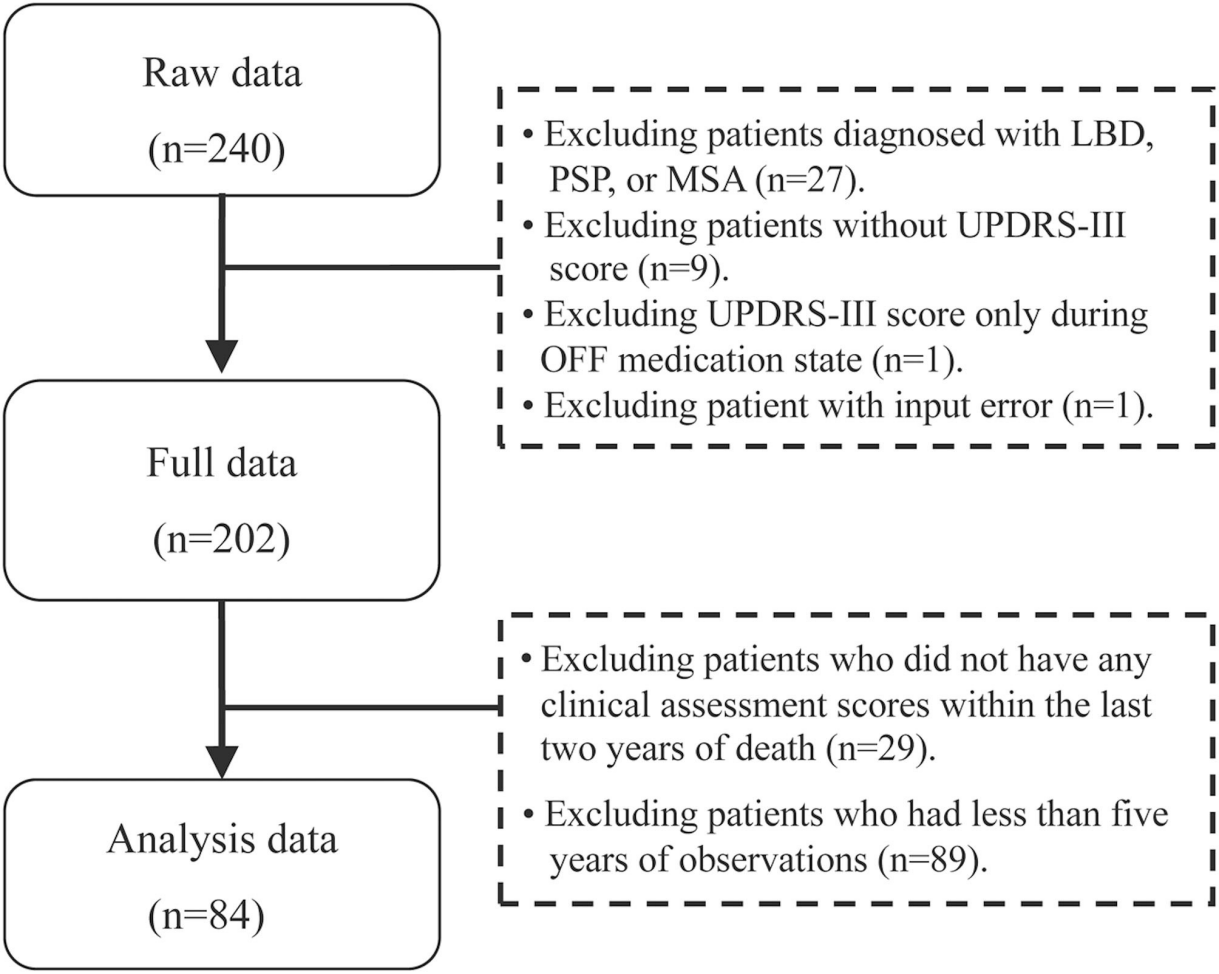
Flowchart of data collection of the patients with Parkinson's disease. Flowchart of final sample of the Parkinson's patients: 84 patients meeting inclusion criteria were analyzed as shown in the flowchart. PD, Parkinson's disease; LBD, Lewy body dementia; MSA, multiple system atrophy; PSP, progressive supranuclear palsy; UPDRS-III, Unified Parkinson's Disease Rating Scale motor score.

The study population included PD patients followed at the Parkinson and Movement Disorder Program (PMDP) at the University of Alberta between 1999 and 2018, and deceased before 2018. Inclusion criteria included: Diagnosis of PD using UK brain bank criteria (14); followed for at least 5 years. Exclusion criteria included: <5 UPDRS III scores from different years, and no UPDRS-III score in the 2 years prior to time of death and those only had off UPDRS scores. Since we were interested in the trajectory patterns approaching death, five or more assessments and scores recorded close to death were deemed necessary. A flowchart shows original data to the final patient sample (Figure 1).

### Chart Review and Data Collection

A retrospective chart review was conducted documenting age at diagnosis and visit, sex, UPDRS-III score at each visit, the year of diagnosis, and two time variables documenting post diagnosis time. One variable was time in years from diagnosis to first UPDRS-III assessment in our clinic, and the other was interval between follow up visits and their first UPDRS-III assessment. Levodopa equivalent daily dose (LEDD) (15) was analyzed at initial visit and at time of death for all patients.

Patients were classified into three age groups according to their age at PD diagnosis, <50, 50–64, and ≥65 years. The endpoints of the study were the trajectories of motor deterioration using UPDRS-III score prior to death, stratified by age group and sex.

To identify patterns of individual trajectory, we first visualized individual UPDRS-III score trajectory using spaghetti plots. We then clustered patients based on their trajectory patterns into different categories. Details of these models can be found in the Appendix in Supplementary Material. If the standard deviations of the UPDRS-III scores were lower than 2.5 across all visits during follow-up, the patients were considered stable. If the UPDRS-III scores were linearly increasing approaching death, the group of patients was categorized into the linear trajectory group. For linear trajectory, we used a mixed effect model to model the average slope of UPDRS-III score with respect to time prior to death, accounting for age group and sex. For patients who had transition points in their trajectories, we grouped the patients by having either one or two transition points in their trajectory. Separate one-knot or two-knot linear model was fitted to each patient's UPDRS III scores. For fitting a one-knot and two-knot model, we require a minimum of 6 and 8 scores from a patient, respectively. To analyze whether UPDRS-III correlated with LEDD, non-parametric correlation with Spearman rho was performed. All statistical analyses were performed using R Statistical Software (16).

### Standard Ethics Approvals

The study was approved by the University of Alberta Health Research Ethics Board (Pro00070137).

## Results

Among 202 deceased PD patients, 84 met inclusion criteria (Figure 1). Demographic and clinical characteristics of the patients are summarized in Table 1. Male to female ratio was 2.42, and 51.2% of the patients were 65 or older at time of diagnosis. The average follow up was 11.4 years among the PD patients, and 64.3% had more than 10 UPDRS-III scores. The median time from PD diagnosis to first assessment was 2 years for the 84 patients (range 0–20 years) (Table 1), 9.5 years (<50), 4 years (50–64), and 1 year for ≥65 years age groups. Among all PD patients, the older the patients were at disease onset, the more likely they were to be followed up early in their disease course. The LEDD increased from first visit (574.6 ± 485.2 mg) to the time of death (864.1 ± 388.7 mg). However, at both time points, patient's LEDD did not correlate with the UPDRS-III score. In addition, the included and excluded decedents were compared (Supplementary Table 1). There was no difference in sex; however, the age of the 84 included patients was younger than the excluded PD patients (*p* < 0.01), and the UPDRS score was 3.4 points higher in the included group at last visit in the included group (*p* < 0.05) (Supplementary Table 1). The excluded decedents had a shorter course from diagnosis to death (*p* < 0.01). Given the shorter course, these decedents did not have sufficient observations to be included in the dataset.

**Table 1:** Demographic and clinical characteristics of the Parkinson's patients analyzed.

**Demographic and clinical characteristics**	***N* (%)**
**Sex**	
Male	59 (70.8)
Female	25 (29.2)
**Time from diagnosis to first UPDRS-III assessment (years)**	
0	21 (25.0)
0– <1	16 (19.1)
1– <5	28 (33.3)
5– <10	12 (14.3)
10– <15	6 (7.1)
>15	1 (1.2)
**Age at diagnosis**	
<50	6 (7.1)
50–64	35 (41.7)
>65	43 (51.2)
**Number of visits**	
5– <10	30 (35.7)
10– <15	35 (41.7)
>15	19 (22.6)

**Figure 2:**
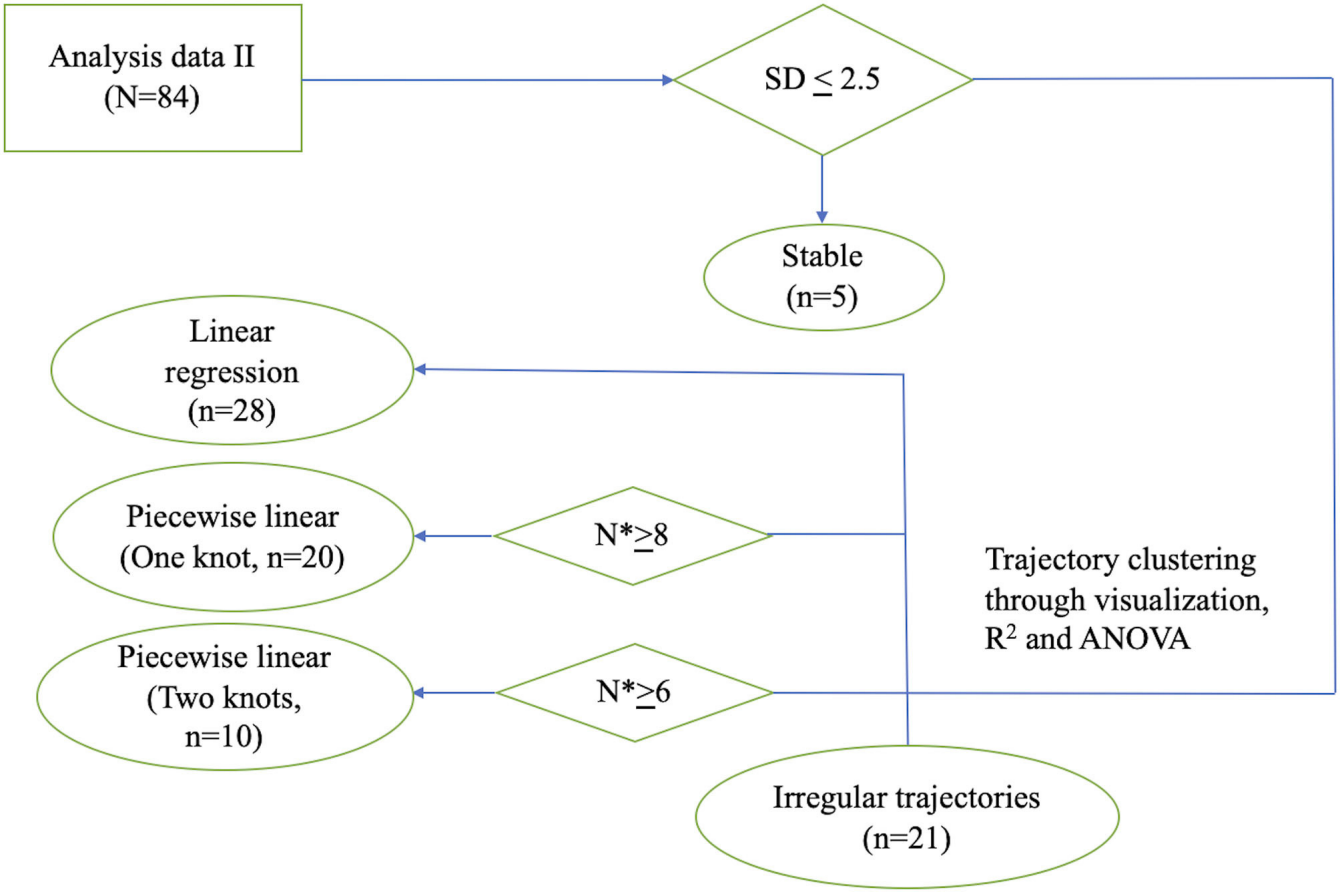
Flowchart illustrating the process of individual model fitting. Flowchart illustrating the process of individual model fitting for the 84 PD patients (*n*: number of patients; N*: number of observations per patient). SD, standard deviation; ANOVA, analysis of variance.

**Figure 3:**
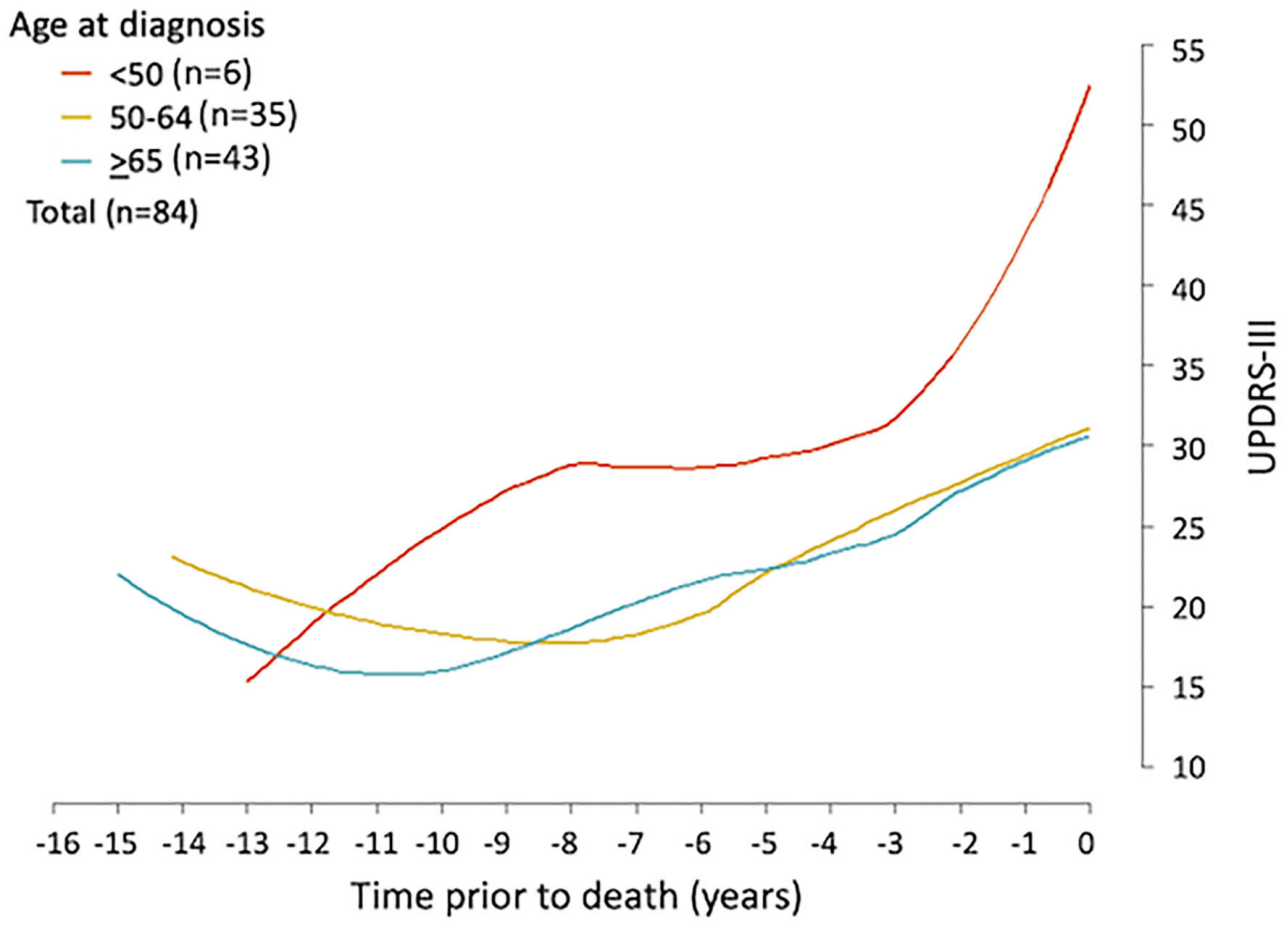
UPDRS-III trajectory prior to death among Parkinson's patients. Loess curves were estimated using UPDRS-III scores from PD patients (*n* = 84) stratified by age at diagnosis, <50, 50–64, and ≥65 years. The figure shows the loess curves of the UPDRS-III scores vs. time for the three age groups combining men and women.

**Figure 4:**
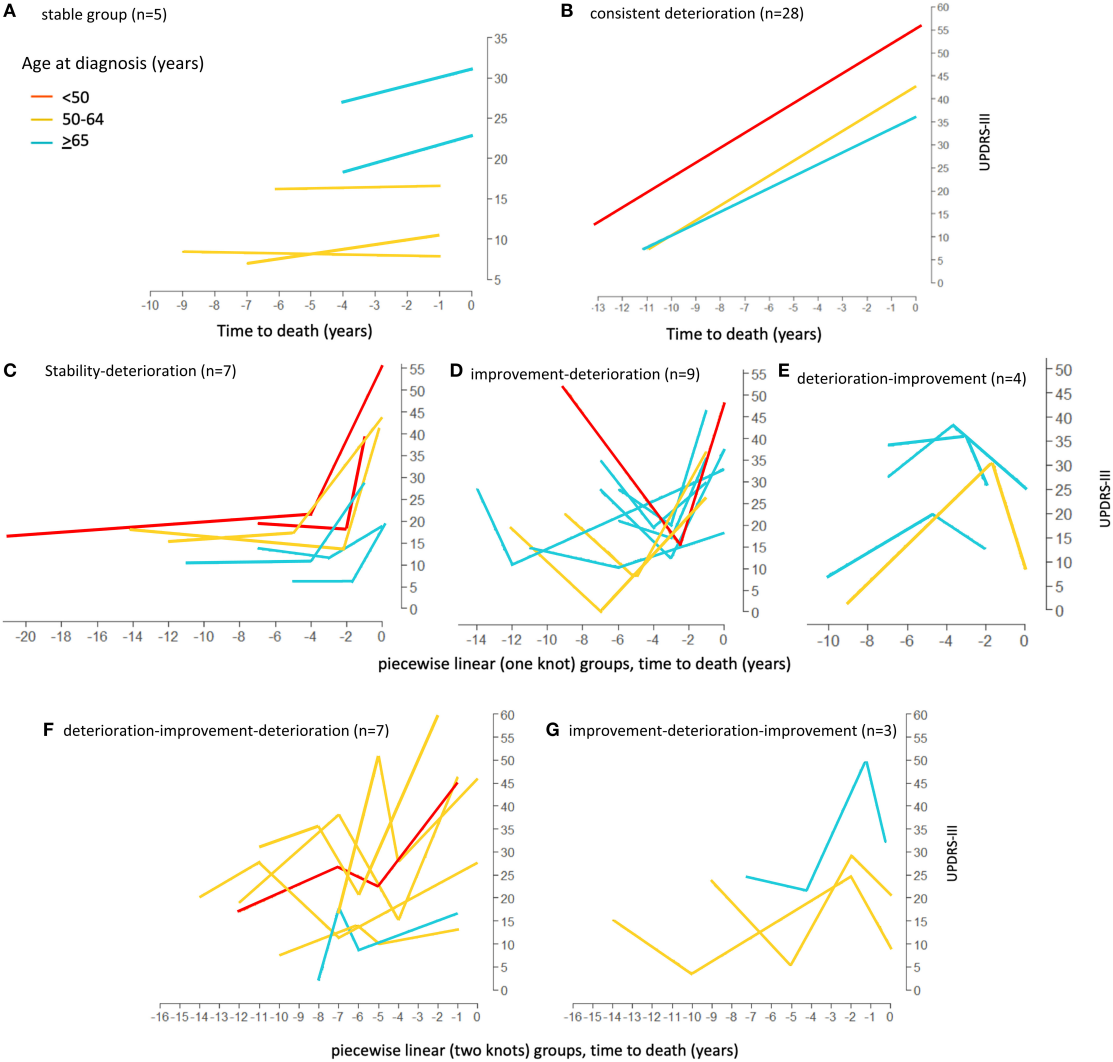
Loess curves of UPDRS-III scores for Parkinson's patients with model fitting. Loess curves were visualized on time prior to death for the 84 Parkinson's patients analyzed. The curve was stratified by age at diagnosis, <50, 50–64, and ≥65 years. The individual model fittings are: **(A)** stable group, *n* = 5. **(B)** linear trend (Consistent deterioration) group (lines represent the average pattern for each age group), *n* = 28. **(C–E)** fitted trajectories for piecewise linear (one knot) groups. **(C)** Stability-Deterioration (*n* = 7); **(D)** Improvement-Deterioration (*n* = 9); and **(E)** Deterioration-Improvement (*n* = 4). **(F,G)** fitted trajectories for piecewise linear (two knots) groups. **(F)** Deterioration-Improvement-Deterioration (*n* = 7); **(G)** Improvement-Deterioration-Improvement (*n* = 3). PD, Parkinson's disease; UPDRS-III, Unified Parkinson's Disease Rating Scale motor score.

Firstly, the spaghetti plots identified patterns of individual UPDRS-III score trajectory, and revealed heterogeneity among PD patients. Based on the trajectory patterns, the patients were grouped into the following categories model: (1) stable, (2) linear, (3) piecewise linear, and (4) irregular (Figure 2). In general, the overall trend of UPDRS-III scores was increasing over time as patients approached death regardless of sex and age of diagnosis (Figure 3). As there is no appreciative difference between men and women, Figure 3 shows the Loess curves of the UPDRS-III scores vs. time for the three age groups combining men and women. Five patients (6%) were in the stable group (Figure 4A). One-third of the PD patients (*n* = 28) belonged to the linear trajectory group (Consistent Deterioration, Figure 4B). Their UPDRS-III scores linearly increased approaching death. In this group, women and men did not have statistically significant different slopes nor did they have different UPDRS-III scores prior to death on average. Age at diagnosis, however, was a predictor for how fast the average UPDRS-III changed. For patients whose age at diagnosis was below 65 years, their average increase in UPDRS-III score was 3.2 per year over their disease course, while the average increase was 2.6 per year for those age at diagnosis was at least 65 years (*p* = 0.023) among the 28 patients in the Consistent Deterioration group. Within the last year of death, the average UPDRS-III scores were 55, 43, and 36 for the three age groups, <50, 50–64, and ≥65, respectively (Figure 4B). However, it should be noted that among the three groups, there were only 6 patients in the <50 group.

There were 20 patients who had one transition point in their UPDRS-III trajectories. Three types of motor trajectories were identified (Figures 4C–E): (A) stable → increase (Stability–Deterioration, *n* = 7); (B) decrease → increase (Improvement–Deterioration, *n* = 9); and (C) increase → decrease (Deterioration–Improvement, *n* = 4). Most of their transitions (85%) occurred between 2 and 5 years prior to death. Another 10 patients had two transition points, seven of whom had an increase → decrease → increase pattern (Deterioration–Improvement–Deterioration, Figure 4F) and three of whom had a decrease → increase → decrease pattern (Improvement–Deterioration–Improvement, Figure 4G). A majority (70%) of patients had their 2nd transitions between 2 and 5 years prior to death.

The remaining 25% of the patients did not fit into any of these above patterns and were classified as having “Undetermined/Irregular” trajectories (*n* = 21). Patients in this group tended to have a later age of onset. Summaries of these patterns were given in Tables 2, 3.

**Table 2:** Trajectory characteristics of the Parkinson patients (time prior to death, *n* = 84).

**(A)** Change in UPDRS-III.				
**Trajectory characteristics**	***n*** **(%)**		**Change in UPDRS-III (points per year), Median (range)**	
Stable^a^	5 (6.0)		NA	
Consistent deterioration	28 (33.3)		3.2^1^, 2.6^2^	
**Piecewise linear (one knot)**				
Stability – Deterioration	7 (8.3)		0.05 (−0.5, 0.3)7.25 (2.9, 21.9)	
Improvement – Deterioration	9 (10.7)		−3.77 (−8.8, −0.9)10.6 (2.2, 18.9)	
Deterioration – Improvement	4 (4.8)		2.85 (0.5, 3.9)−8.59 (−17.1 −5.1)	
**Piecewise linear (two knot)**				
Deterioration – Improvement – Deterioration	7 (8.3)		2.5 (1.5, 16.9)−9.0 (−39.9, −4.1)10.9 (5.0, 27.5)	
Improvement – Deterioration – Improvement	3 (3.6)		−2.9 (−1.0, −4.5)10.5 (5.6, 12.5)−12.25 (−27.6, −10.2)	
Irregular/Undetermined	21 (25.0)		NA	
**(B)** Transition time, time prior to death (years).				
**Trajectory characteristics**	***n*** **(%)**	**Transition 1** **Median (range)**	**Transition 2** **Median (range)**	**Age at diagnosis (years)** **Median (range)**
Stable^a^	5 (6.0)	NA	NA	60.5 (56, 75)
Consistent deterioration	28 (33.3)	NA	NA	68 (43, 80)
**Piecewise linear (one knot)**				
Stability – Deterioration	7 (8.3)	−3 (−5, −1.7)	NA	63 (47, 70)
Improvement – Deterioration	9 (10.7)	−4 (−12, −2.5)	NA	70 (48, 81)
Deterioration – Improvement	4 (4.8)	−3.35 (−4.7, −1.7)	NA	67 (61, 75)
**Piecewise linear (two knots)**				
Deterioration – Improvement – Deterioration	7 (8.3)	−7 (−11, −5)	−5 (−7, −4)	56 (45, 67)
Improvement – Deterioration – Improvement	3 (3.6)	−5 (−10, −4.3)	−2 (−2, −1.3)	64 (61, 73)
Irregular/Undetermined	21 (25.0)	NA	NA	63 (52, 76)

a *Patients with standard deviation <2.5*;

1 *Fixed effects slope for age group (<50 and 50–64 years)*;

2 *fixed effects slope for age group (≥65 years)*.

**Table 3:** Summary of the trajectory types in relation to sex and age of onset of Parkinson's disease.

**Trajectory characteristics**		**Sex**, ***n*****(col%)**		**Total** ***n* (col%)**
		**Male**	**Female**	
Stable		3 (5.1)	2 (8.0)	5 (6.0)
Consistent deterioration		19 (32.3)	9 (36.0)	28 (33.3)
Stability – Deterioration		5 (8.5)	2 (8.0)	7 (8.3)
Improvement – Deterioration		5 (8.5)	4 (16.0)	9 (10.7)
Deterioration – Improvement		3 (5.1)	1 (4.0)	4 (4.8)
Deterioration – Improvement – Deterioration		6 (10.2)	1 (4.0)	7 (8.3)
Improvement – Deterioration – Improvement		2 (3.4)	1 (4.0)	3 (3.6)
Irregular/Undetermined		16 (27.1)	5 (20.0)	21 (25.0)
Total *n* (row %)		59 (70.2)	25 (29.8)	84 (100)
**Trajectory characteristics**	**Age at diagnosis** ***n*** **(col%)**			**Total** ***n*** **(col%)**
	**<50**	**50–64**	**≥65**	
Stable	0 (0)	3 (8.6)	2 (4.6)	5 (6.0)
Consistent deterioration	2 (33.3)	8 (22.8)	18 (41.9)	28 (33.3)
Stability – Deterioration	2 (33.3)	2 (5.7)	3 (7.0)	7 (8.3)
Improvement – Deterioration	1 (16.7)	2 (5.7)	6 (14.0)	9 (10.7)
Deterioration – Improvement	0 (0)	2 (5.0)	2 (5.4)	4 (4.8)
Deterioration – Improvement – Deterioration	1 (16.7)	5 (14.3)	1 (2.3)	7 (8.3)
Improvement – Deterioration – Improvement	0 (0)	2 (5.7)	1 (2.3)	3 (3.6)
Irregular/Undetermined	0 (0)	12 (34.3)	9 (20.9)	21 (25.0)
Total *n* (row %)	6 (7.1)	35 (41.7)	43 (51.2)	84 (100)

There was no difference in post diagnosis life expectancy between men and woman in our study (Table 4). In contrast, age of PD diagnosis was associated with survival time. Patients younger than 50 years old at diagnosis had a median survival of 24 years, compared to 8 years in patients >65 years old (Table 4).

**Table 4:** Survival time post diagnosis for the Parkinson's patients.

**Characteristics**	***n* (%)**	**Median (IQR)**	**Range**
**Sex**			
Male	59 (70.2)	11 (8–17)	(5–30)
Female	25 (29.8)	13 (9–18)	(5–22)
**Age at diagnosis groups (years)**			
<50	6 (7.1)	24 (19–27)	(18–30)
50–64	35 (41.7)	15 (12–18)	(6–26)
≥65	43 (51.2)	10 (7–11)	(5–20)

*The survival time of the parkinsonian syndromes in relation to sex and age of the patients. Survival time, survival from time of diagnosis in years. IQR, interquartile range*.

## Discussion

Our retrospective study of PD to death revealed different trajectory patterns (e.g., Consistent Deterioration, Stability-Deterioration, Improvement-Deterioration, Deterioration-Improvement-Deterioration) as assessed by the UPDRS-III. Across the trajectory patterns, the trend of “Decrease” in UPDRS-III before the “Increase” might have been due to initial response to medication initiation or adjustment, and possible introduction of physio/occupation therapy or other multidisciplinary care interventions, resulting in UPDRS III improvement. As PD progresses to late stage, disability progression may relate to a loss of compensatory abilities, widespread Lewy bodies and coexistent pathologies (i.e., vascular, plaques and tangles) (17, 18). Only a small percentage of patients had stable motor function (6%).

Over time, UPDRS-III scores showed a steep increase toward death in many patients. The “terminal decline” in PD could be attributed to changes in levodopa intake (i.e., dysphagia, necessary adjustments due to neuropsychiatric complications, gastrointestinal complications, hospitalization, and nursing home placement with less individualized care) and pharmacodynamic changes with loss of responsiveness to dopaminergic medications. In non-PD elderly, impaired motor function and faster rate of motor decline were associated with increased mortality (19). Lunney et al. has summarized terminal decline into four groups with different duration and shape in the aging population (20): sudden death; terminally-ill (rapid decline until death, i.e., in cancer); organ failure (gradual decline with frequent episodic acute exacerbations); and frailty (chronic disease with slow and gradual decline). Our cohort demonstrated variable patterns that contrast with non-PD elderly including a terminal decline 2–5 years prior to death.

Our findings of variable patterns of motor impairment trajectories confirm clinicians', patients', and families' experience that PD has many presentations, but terminal decline in motor function is common. Rather than the generic, “every patient is different” advice commonly received by PD patients and families, our results may allow clinicians to provide more nuanced information. The pathophysiologic basis for the different trajectories is not clear as we do not have radiographic, pathologic or genetic information. However, our results can be taken as clinical evidence that PD is potentially a spectrum of illnesses rather than a uniform entity and that identifiable patterns do exist (21). Further, our results demonstrate a significant age difference across all trajectory types (Table 4), with younger patients having longer survival. This is also valuable information for clinicians, patients and families. Given the difference between those diagnosed prior to age 50 and those diagnosed after 65 years of age, this is significant prognostic information for patients.

Details on PD motor progression were not well-documented with validated rating scales in the pre-levodopa era (22, 23). The introduction of dopaminergic agents improved motor function and disability in PD, but did not translate to reduced mortality in a 10-year multicentered study (24). These investigators reported that if advanced PD was defined by the appearance of axial symptoms and dementia, both bromocriptine and levodopa groups progressed at a similar rate. In addition, current medical and surgical therapies have not been shown to significantly alter the progression of the underlying neurodegeneration process in PD (4, 5). PD trajectories are complicated by phenotypic heterogeneity, diagnostic inaccuracy, and confounding factors including age and comorbidities (25). Further, recent genetic advances bring into question whether PD as the phenotype is indeed a single illness (21). Many placebo-controlled trials defined the rates of progression of motor dysfunction using the Unified Parkinson's Disease Rating Scale (UPDRS II and III) within the first 2–5 years of PD diagnosis (26–28). The rate of progression decreases with longer follow-up of 4 (29) and 8 years (30). This is consistent with previous clinical cross-sectional studies (23, 31, 32). The non-linear progression of PD motor impairment with steeper declines earlier in the disease may be due to an exponential decline of neuronal cell counts in the substantial nigra (33).

PD increases mortality compared to age-matched non-PD (34, 35). It is the 14th leading cause of death in the US (36). In a large population-based study, Beyer et al. indicated that age, UPDRS scores, and Hoehn and Yahr stage at baseline were greater in those who died during the follow-up period compared to the survivors (35). However, no longitudinal changes were collected during the follow-up. Our work provides importance evidence that terminal decline is a feature of PD, with later age of diagnosis associated with much shorter survival (10 years with diagnosis after 65 years of age vs. 24 years with diagnosis before age 50) (Table 4). This is valuable prognostic information for clinicians, patients, and families. In late stage disease, motor features seemed to become less responsive to dopaminergic therapy. Therefore, for those over 65 years of age at time of diagnosis, consideration of early implementation of palliative care principles of care would be appropriate. In the setting of motor complications, earlier rather than later DBS may be indicated given the relatively less progressive and long course especially in young onset (diagnosis before age 50) patient population (37, 38).

We did not observe any differences in disease trajectory between men and women as patients approached death. Previous longitudinal and cross-sectional studies of sex differences in PD progression yielded mixed findings. A longitudinal, observational study with 4,679 PD patients indicated that no significant differences between men and women were observed after 1 year of follow-up (39). However, baseline characteristics were different with women being significantly older than male participants in their study. A large clinical trial found no difference between male and female PD patients who were on similar treatment regimens before enrollment during early stages of disease (40). In contrast, faster clinical decline was reported in men compared to women in another study (41). However, baseline clinical features between male and female patients were not analyzed in this study.

Consistent with our findings that patients older than 65 had shorter survival time, a previous systematic review using cluster analysis identified PD subtypes: young age (≤40 years old) at onset with slow disease progression, and old age (≥70 years old) at onset with rapid disease progression (13). A long term follow up study showed an increase hazard ratio for mortality of 1.40 for every 10-year increase in age (42). Similarly, older age at onset was a predictive factor for more rapid motor progression, nursing home placement, and shorter survival time (43), and was associated with progression of non-levodopa-responsive symptoms (44).

In our study, within the Consistent Deterioration group (Figure 4B, *n* = 28), age at diagnosis was associated with terminal UPDRS-III score. The older patients (≥65) had a lower UPDRS-III score toward death. However, it is hard to conclude that this observation represents the true natural history since it is only restricted to the Consistent Deterioration group with linear trajectory, as well as restricted to the end of life period instead of the entire survival period. In addition, we have only 6 patients belonging to this group whose age of onset was <50 years. It should be noted that, in young onset patients, we have missed years of follow up between age of onset and age at first assessment. The six individuals showed linear trajectory for the duration of analysis. However, to study the true pattern of progression in the patients with young age of onset, further studies, using larger sample sizes, are needed to use nature age as the time axis. Therefore, for patients diagnosed at younger age, the pattern of trajectory could potentially change to a different profile.

For those living with PD, planning for future needs especially in late stage is important. Our identification of terminal motor decline, similar to that in non-PD elderly, can provide a signpost for clinicians to inform patients and especially family members of the arrival of a new stage of illness. Terminal decline of UPDRS III can provide families with a sign that is easy for them to grasp and appreciate. Clinicians who identify terminal decline in their patients can use this information to guide a discussion regarding patient and caregiver needs and the potential benefits of palliative care involvement. Activating palliative care can engage the holistic philosophy that may relieve burdensome symptoms that accompany terminal decline such as pain, shortness of breath, caregiver burden, dysphagia, and delirium (2). Furthermore, this information can help counsel patients with advanced disease to dispel the notion that PD has a constant rate of motor deterioration and educate about realistic expectations for the future. Our results can act as a trigger to help engage patients and caregivers in multidimensional shared decision-making discussions and make well-informed and thoughtful care decisions based on PD progression. The novel statistical approach to analyze disease trajectory resulting in distinct patterns is also clinically relevant. The modeling approach takes into account within patient score correlation, allowing teasing out the effect of age group and sex on the linear trend of the longitudinal scores. Due to limited sample size in some patterns of trajectory, we were unable to use statistical models to identify when change point(s) occur prior to death on average. We described and summarized our observation of individual trajectories instead (Figures 4A,C–G) and Tables 2, 3.

There are some limitations of the current study. Since the study was a retrospective chart review, other aspects of importance in advanced PD such as cognitive, other non-motor features, comorbidities and quality of life measures were not examined. Nevertheless, the UPDRS-III is used widely to reflect PD motor disability and is a main outcomes measure in many symptomatic trials (45, 46). However, it is possible that the UPDRS-III does not completely reflect true functional status of patients or progression rate at higher levels of disease severity. UPDRS-III scores may not be reliable in advanced disease due to a ceiling effect (47). We did not perform subgroup UPDRS III score analysis (tremor-dominant or PIGD) as previous studies have done (27, 28, 48, 49). Due to the long-time frame of data collection, the new MDS-UPDRS was not used. Our dataset was small as we limited ourselves to decedents with “complete” data. We acknowledge that additional patterns of change may be possible with large sample size. It is also important to note that 25% of our PD patients could not be classified into a pattern – this may be due to inability of patients to fully participate in examination or other factors not controlled for in a clinical setting. Our criteria for inclusion could have excluded older patients who were unable to be assessed in the clinic within 2 years prior to death, given the age difference between the included and excluded decedents. Similarly, with lack of the UPDRS-III in the final 2 years, the excluded group had a slightly lower UPDRS-III score at last assessment. In addition, in order to analyze the trajectory, we excluded the patients with <5 years of visit, which likely led to the shorter disease duration in the excluded group. However, this does not refute the observed patterns in the terminal motor trajectory in the PD patients with more complete data.

Future prospective studies of motor progression, non-motor symptoms, cognitive and neurobehavioral symptoms and the impact of comorbidities are needed to better characterize the totality of PD progression. While our retrospective study has provided a framework for counseling PD patients and caregivers, future prospective studies including reliable metrics that assess global function, including motor, non-motor, and activities of daily living, can further categorize disease trajectory and provide more accurate and holistic information.

Despite these limitations, our results outline four main types of motor progression in the years leading to death (Consistent Deterioration, Stability-Deterioration, Improvement-Deterioration, Deterioration-Improvement-Deterioration) and that, regardless of motor progression, terminal motor decline with a steep increase in UPDRS III was seen in many patients approaching death. Those with diagnosis after age 65 years had shorter survival times. Our study provides knowledge of dominant trajectory types in PD that can help clinicians understand their patients' course of illness. This information can help counsel patients with advanced disease to identify triggers of declining function and potentially may be used for hospice enrolment criteria or involvement of palliative care.

## Data Availability Statement

The raw data supporting the conclusions of this article will be made available by the authors, without undue reservation.

## Ethics Statement

The studies involving human participants were reviewed and approved by University of Alberta Health Research Ethics Board. Written informed consent for participation was not required for this study in accordance with the national legislation and the institutional requirements.

## Author Contributions

SP: statistical analysis – review and critique and manuscript – writing of the first draft and review and critique. JM: research project – conception, organization, execution, statistical analysis – design and review and critique, and manuscript – review and critique. XF: research project – execution and statistical analysis – execution and review and critique. RC: research project – conception, organization, execution, statistical analysis – review and critique, and manuscript – review and critique. TS: research project – execution, statistical analysis – execution and review, and manuscript – review and critique. YY: statistical analysis – design and review and critique and manuscript – review and critique. FB: research project – conception and execution, statistical analysis – review and critique, and manuscript – writing of the first draft and review and critique. All authors contributed to the article and approved the submitted version.

## Conflict of Interest

The authors declare that the research was conducted in the absence of any commercial or financial relationships that could be construed as a potential conflict of interest.

## Publisher's Note

All claims expressed in this article are solely those of the authors and do not necessarily represent those of their affiliated organizations, or those of the publisher, the editors and the reviewers. Any product that may be evaluated in this article, or claim that may be made by its manufacturer, is not guaranteed or endorsed by the publisher.

## References

[1] Kluger BM Katz M Galifianakis N Pantilat SZ Kutner JS Sillau S. Does outpatient palliative care improve patient-centered outcomes in Parkinson's disease: rationale, design, and implementation of a pragmatic comparative effectiveness trial. Contemp Clin Trials. (2019) 79:28–36. 10.1016/j.cct.2019.02.005 30779960

[2] Kluger BM Miyasaki J Katz M Galifianakis N Hall K Pantilat S. Comparison of integrated outpatient palliative care with standard care in patients with parkinson disease and related disorders: a randomized clinical trial. JAMA Neurol. (2020) 77:551–60. 10.1001/jamaneurol.2019.4992 32040141 PMC7042842

[3] Macchi ZA Koljack CE Miyasaki JM Katz M Galifianakis N Prizer LP. Patient and caregiver characteristics associated with caregiver burden in Parkinson's disease: a palliative care approach. Ann Palliat Med. (2020) 9:S24–33. 10.21037/apm.2019.10.01 31735048

[4] Poewe W Mahlknecht P. The clinical progression of Parkinson's disease. Parkinsonism Relat Disord. (2009) 15:S28–32. 10.1016/S1353-8020(09)70831-4 20123553

[5] Poewe W. The natural history of Parkinson's disease. J Neurol. (2006) 253:VII2–6. 10.1007/s00415-006-7002-7 17131223

[6] Ba F Obaid M Wieler M Camicioli R Martin WR. Parkinson disease: the relationship between non-motor symptoms and motor phenotype. Can J Neurol Sci. (2016) 43:261–7. 10.1017/cjn.2015.328 26949837

[7] Chaudhuri KR Healy DG Schapira AH. Non-motor symptoms of Parkinson's disease: diagnosis and management. Lancet Neurol. (2006) 5:235–45. 10.1016/S1474-4422(06)70373-8 16488379

[8] Fahn S Jankovic J Hallett M. Principles and Practice of Movement Disorders. 2nd ed. Edinburgh: Elsevier Saunders (2011). 10.1016/B978-1-4377-2369-4.00025-1

[9] de Lau LM Breteler MM. Epidemiology of Parkinson's disease. Lancet Neurol. (2006) 5:525–35. 10.1016/S1474-4422(06)70471-9 16713924

[10] Daroff RB Jankovic J Mazziotta JC Pomeroy SL Bradley WG. Bradley's Neurology in Clinical Practice. 7th ed. Cambridge, MA: Elsevier (2016).

[11] Wenning GK Ebersbach G Verny M Chaudhuri KR Jellinger K McKee A. Progression of falls in postmortem-confirmed parkinsonian disorders. Mov Disord. (1999) 14:947–50. 10.1002/1531-8257(199911)14:6<947::AID-MDS1006>3.0.CO;2-O 10584668

[12] Müller J Wenning GK Verny M McKee A Chaudhuri KR Jellinger K. Progression of dysarthria and dysphagia in postmortem-confirmed parkinsonian disorders. Arch Neurol. (2001) 58:259–64. 10.1001/archneur.58.2.259 11176964

[13] Jankovic J McDermott M Carter J Gauthier S Goetz C Golbe L. Variable expression of Parkinson's disease: a base-line analysis of the DATATOP cohort. The Parkinson Study Group. Neurology. (1990) 40:1529–34. 10.1212/WNL.40.10.1529 2215943

[14] Hughes AJ Daniel SE Ben-Shlomo Y Lees AJ. The accuracy of diagnosis of parkinsonian syndromes in a specialist movement disorder service. Brain. (2002) 125:861–70. 10.1093/brain/awf080 11912118

[15] Tomlinson CL Stowe R Patel S Rick C Gray R Clarke CE. Systematic review of levodopa dose equivalency reporting in Parkinson's disease. Mov Disord. (2010) 25:2649–53. 10.1002/mds.23429 21069833

[16] Team RC. R: A Language and Environment for Statistical Computing. R Foundation for Statistical Computing, Vienna, Austria (2017).

[17] Braak H Del Tredici K. Neuropathological staging of brain pathology in sporadic Parkinson's disease: separating the wheat from the chaff. J Parkinsons Dis. (2017) 7:S71–85. 10.3233/JPD-179001 28282810 PMC5345633

[18] Sierra M Gelpi E Marti MJ Compta Y. Lewy- and Alzheimer-type pathologies in midbrain and cerebellum across the Lewy body disorders spectrum. Neuropathol Appl Neurobiol. (2016) 42:451–62. 10.1111/nan.12308 26810462

[19] Buchman AS Wilson RS Boyle PA Bienias JL Bennett DA. Change in motor function and risk of mortality in older persons. J Am Geriatr Soc. (2007) 55:11–9. 10.1111/j.1532-5415.2006.01032.x 17233680

[20] Lunney JR Lynn J Hogan C. Profiles of older medicare decedents. J Am Geriatr Soc. (2002) 50:1108–12. 10.1046/j.1532-5415.2002.50268.x 12110073

[21] Poewe W Seppi K Tanner CM Halliday GM Brundin P Volkmann J. Parkinson disease. Nat Rev Dis Primers. (2017) 3:17013. 10.1038/nrdp.2017.13 28332488

[22] Hoehn MM Yahr MD. Parkinsonism: onset, progression and mortality. Neurology. (1967) 17:427–42. 10.1212/WNL.17.5.427 6067254

[23] Goetz CG Tanner CM Shannon KM. Progression of Parkinson's disease without levodopa. Neurology. (1987) 37:695–8. 10.1212/WNL.37.4.695 3561783

[24] Hely MA Morris JGL Traficante R Reid WGJ O'Sullivan DJ Williamson PM. The Sydney multicentre study of Parkinson's disease: progression and mortality at 10 years. J Neurol Neurosurg Psychiatry. (1999) 67:300–7. 10.1136/jnnp.67.3.300 10449550 PMC1736543

[25] Coughlin DG Hurtig HI Irwin DJ. Pathological influences on clinical heterogeneity in lewy body diseases. Mov Disord. (2020) 35:5–19. 10.1002/mds.27867 31660655 PMC7233798

[26] Parkinson Study G. Effects of tocopherol and deprenyl on the progression of disability in early Parkinson's disease. N Engl J Med. (1993) 328:176–83. 10.1056/NEJM199301213280305 8417384

[27] Olanow CW Hauser RA Jankovic J Langston W Lang A Poewe W. A randomized, double-blind, placebo-controlled, delayed start study to assess rasagiline as a disease modifying therapy in Parkinson's disease (the ADAGIO study): rationale, design, and baseline characteristics. Mov Disord. (2008) 23:2194–201. 10.1002/mds.22218 18932271

[28] Fahn S Oakes D Shoulson I Kieburtz K Rudolph A Lang A. Levodopa and the progression of Parkinson's disease. N Engl J Med. (2004) 351:2498–508. 10.1056/NEJMoa033447 15590952

[29] Goetz CG Stebbins GT Blasucci LM. Differential progression of motor impairment in levodopa-treated Parkinson's disease. Mov Disord. (2000) 15:479–84. 10.1002/1531-8257(200005)15:3<479::AID-MDS1009>3.0.CO;2-P 10830412

[30] Louis ED Tang MX Cote L Alfaro B Mejia H Marder K. Progression of parkinsonian signs in Parkinson disease. Arch Neurol. (1999) 56:334–7. 10.1001/archneur.56.3.334 10190824

[31] Poewe WH Wenning GK. The natural history of Parkinson's disease. Ann Neurol. (1998) 44:S1–9. 10.1002/ana.410440703 9749568

[32] Poewe WH Wenning GK. The natural history of Parkinson's disease. Neurology. (1996) 47:S146–52. 10.1212/WNL.47.6_Suppl_3.146S 8959983

[33] Fearnley JM Lees AJ. Ageing and Parkinson's disease: substantia nigra regional selectivity. Brain. (1991) 114:2283–301. 10.1093/brain/114.5.2283 1933245

[34] Coelho M Ferreira JJ. Late-stage Parkinson disease. Nat Rev Neurol. (2012) 8:435–42. 10.1038/nrneurol.2012.126 22777251

[35] Beyer MK Herlofson K Arsland D Larsen JP. Causes of death in a community-based study of Parkinson's disease. Acta Neurol Scand. (2001) 103:7–11. 10.1034/j.1600-0404.2001.00191.x 11153892

[36] Dorsey ER George BP Leff B Willis AW. The coming crisis: obtaining care for the growing burden of neurodegenerative conditions. Neurology. (2013) 80:1989–96. 10.1212/WNL.0b013e318293e2ce 23616157 PMC3716348

[37] Deuschl G Schade-Brittinger C Agid Y Group ES. Neurostimulation for Parkinson's disease with early motor complications. N Engl J Med. (2013) 368:2038. 10.1056/NEJMc1303485 23697520

[38] Kleiner-Fisman G Herzog J Fisman DN Tamma F Lyons KE Pahwa R. Subthalamic nucleus deep brain stimulation: summary and meta-analysis of outcomes. Mov Disord. (2006) 21:S290–304. 10.1002/mds.20962 16892449

[39] Dahodwala N Pei Q Schmidt P. Sex Differences in the clinical progression of Parkinson's Disease. J Obstet Gynecol Neonatal Nurs. (2016) 45:749–56. 10.1016/j.jogn.2016.05.002 27444842 PMC5021611

[40] Augustine EF Perez A Dhall R Umeh CC Videnovic A Cambi F. Sex differences in clinical features of early, treated Parkinson's disease. PLoS ONE. (2015) 10:e0133002. 10.1371/journal.pone.0133002 26171861 PMC4501841

[41] Jankovic J Kapadia AS. Functional decline in Parkinson disease. Arch Neurol. (2001) 58:1611–5. 10.1001/archneur.58.10.1611 11594919

[42] Forsaa EB Larsen JP Wentzel-Larsen T Alves G. What predicts mortality in Parkinson disease?: a prospective population-based long-term study. Neurology. (2010) 75:1270–6. 10.1212/WNL.0b013e3181f61311 20921512

[43] Suchowersky O Reich S Perlmutter J Zesiewicz T Gronseth G Weiner WJ. Practice Parameter: diagnosis and prognosis of new onset Parkinson disease (an evidence-based review): report of the Quality Standards Subcommittee of the American Academy of Neurology. Neurology. (2006) 66:968–75. 10.1212/01.wnl.0000215437.80053.d0 16606907

[44] Velseboer DC Broeders M Post B van Geloven N Speelman JD Schmand B. Prognostic factors of motor impairment, disability, and quality of life in newly diagnosed PD. Neurology. (2013) 80:627–33. 10.1212/WNL.0b013e318281cc99 23345637

[45] Marks WJ Jr Bartus RT Siffert J Davis CS Lozano A Boulis N. Gene delivery of AAV2-neurturin for Parkinson's disease: a double-blind, randomised, controlled trial. Lancet Neurol. (2010) 9:1164–72. 10.1016/S1474-4422(10)70254-4 20970382

[46] Brys M Fox MD Agarwal S Biagioni M Dacpano G Kumar P. Multifocal repetitive TMS for motor and mood symptoms of Parkinson disease: a randomized trial. Neurology. (2016) 87:1907–15. 10.1212/WNL.0000000000003279 27708129 PMC5100715

[47] Lewis MM Harkins E Lee EY Stetter C Snyder B Corson T. Clinical progression of Parkinson's disease: insights from the NINDS Common Data Elements. J Parkinsons Dis. (2020) 10:1075–85. 10.3233/JPD-201932 32538866 PMC8177750

[48] Schrag A Dodel R Spottke A Bornschein B Siebert U Quinn NP. Rate of clinical progression in Parkinson's disease. A prospective study. Mov Disord. (2007) 22:938–45. 10.1002/mds.21429 17415791

[49] Lee CS Schulzer M Mak EK Snow BJ Tsui JK Calne S. Clinical observations on the rate of progression of idiopathic parkinsonism. Brain. (1994) 117:501–7. 10.1093/brain/117.3.501 8032860

