# Supplementary material for: The Trajectory of Motor Deterioration to Death in Parkinson's Disease
Source: Front Neurol. 2021 Aug 18;12:670567. doi: 10.3389/fneur.2021.670567 (PMC8416311; doi:10.3389/fneur.2021.670567)
Supplement: Supplementary file 1 [file Table_1.docx]

Supplementary Material

# Supplementary Material

**Appendix:**

One-knot model: $Y_{ij}=\beta_{0}+\beta_{1}t_{ij}+\beta_{2}{(t_{ij}-\xi)}_{+}+\varepsilon_{ij}$, j = 1, 2,…$k_{i}$

Two-knot model: $Y_{ij}=\beta_{0}+\beta_{1}t_{ij}+\beta_{2}{(t_{ij}-\xi_{i1})}_{+}+\beta_{3}{(t_{ij}-\xi_{i2})}_{+}+\varepsilon_{ij}$, j = 1, 2,…$k_{i}$

where Y is the UPDRS-III score, *t* is the year prior to death, $\xi_{1}, \xi_{2} \in(0, t_{1})$ are transition points determined by visual inspection, ${(t_{ij}-\xi)}_{+}$takes on value 0 for $t_{ij}$ < $\xi$ and takes on value $\left( t_{ij}-\xi\right)$ for $t_{ij}$ ≥ $\xi$, j indexes the visit number for a patient, ranging from 1 to $k_{i}$. $k_{i}$ is the total number of yearly visit for the i^th^ patient. Note that *t_1_* indexes the year of a patient’s first visit with a UPDRS-III score and is the furthest from that patient’s date of death.

**Supplemental table 1**: Comparison of the baseline demographic feature of the included Parkinson’s decedents with the total group and the excluded patients.

|  | **Age (year)**  Median (range) | **Sex**  Male Female  n (%) n (%) | | **Duration of disease (year)**  Median (range) | **Motor severity^1^**  Median (range) |
| --- | --- | --- | --- | --- | --- |
| Total PD patients (n=202) | 67.4  68 (28, 90) | 129 (63.9) | 73 (36.1) | 10.5 (0, 47) | 30 (5, 60) |
| Included PD patients (n=84) | 64.6^*^  65 (43, 81) | 59 (70.2) | 25 (29.8) | 12 (5, 30)^*^ | 31 (6.5, 59)^*^ |
| Excluded PD patients (n=118) | 69.4  71 (28, 90) | 70 (59.3) | 48 (40.7) | 8 (0, 47) | 28.4 (5, 60) |

^1^Motor severity was terminal decline was represented by the last UDPRS-III prior to death from clinical assessment.

*, P<0.05, comparing between the included Parkinson patients with excluded patients. PD, Parkinson’s disease.
